# Development of a Novel Trap for the Collection of Black Flies of the *Simulium ochraceum* Complex

**DOI:** 10.1371/journal.pone.0076814

**Published:** 2013-10-07

**Authors:** Mario A. Rodríguez-Pérez, Monsuru A. Adeleke, Nathan D. Burkett-Cadena, Javier A. Garza-Hernández, Filiberto Reyes-Villanueva, Eddie W. Cupp, Laurent Toé, Mario C. Salinas-Carmona, Américo D. Rodríguez-Ramírez, Charles R. Katholi, Thomas R. Unnasch

**Affiliations:** 1 Centro de Biotecnología Genómica, Instituto Politécnico Nacional, Reynosa, Tamaulipas, México; 2 Facultad de Medicina, Universidad Autónoma de Nuevo León, Monterrey, Nuevo León, México; 3 Department of Biological Sciences, Osun State University, Osogbo, Nigeria; 4 Global Health Infectious Disease Research Program, Department of Global Health, University of South Florida, Tampa, Florida, United States of America; 5 Department of Entomology and Plant Pathology, Auburn University, Auburn, Alabama, United States of America; 6 Multidisease Surveillance Centre, World Health Organization, Ouagadougou, Burkina Faso; 7 Centro Regional de Investigación en Salud Pública, Instituto Nacional de Salud Pública, Tapachula, Chiapas, México; 8 Department of Biostatistics, University of Alabama at Birmingham, Birmingham, Alabama, United States of America; University of Tours, France

## Abstract

**Background:**

Human landing collections are currently the standard method for collecting onchocerciasis vectors in Africa and Latin America. As part of the efforts to develop a trap to replace human landing collections for the monitoring and surveillance of onchocerciasis transmission, comprehensive evaluations of several trap types were conducted to assess their ability to collect *Simulium* *ochraceum* sensu lato, one of the principal vectors of *Onchocerca volvulus* in Latin America.

**Methodology/Principal Findings:**

Diverse trap designs with numerous modifications and bait variations were evaluated for their abilities to collect *S. Ochraceum* s.l. females. These traps targeted mostly host seeking flies. A novel trap dubbed the “Esperanza window trap” showed particular promise over other designs. When baited with CO_2_ and BG-lure (a synthetic blend of human odor components) a pair of Esperanza window traps collected numbers of *S. Ochraceum* s.l. females similar to those collected by a team of vector collectors.

**Conclusions/Significance:**

The Esperanza window trap, when baited with chemical lures and CO_2_ can be used to collect epidemiologically significant numbers of *Simulium* *ochraceum* s.l., potentially serving as a replacement for human landing collections for evaluation of the transmission of *O. volvulus*.

## Introduction

Onchocerciasis (river blindness) is caused by chronic infection with *Onchocerca volvulus*, a filarial nematode that is transmitted by *Simulium* spp. (Diptera: Simuliidae). The disease constitutes a serious public health concern and an enormous source of socio-economic loss in many developing countries, most severely in sub-Saharan Africa and, to a lesser extent, Latin America [1-5]. The current strategy for the elimination of onchocerciasis relies on mass treatment of endemic communities with ivermectin. A variety of treatment regimens, including quarterly, semi-annual and annual treatment have proven effective in focally interrupting transmission and eliminating the parasite in Latin America and in isolated foci in Africa over different time frames [6-12]. High coverage (≥ 85% of eligible persons) community-wide treatment of residents for a minimum of 15 years is believed to be sufficient to reduce the load of microfilariae in human hosts below the threshold that can sustain transmission by black fly vectors, thus locally eliminating the infection [7].

The elimination guidelines set forth by the Onchocerciasis Elimination Program for the Americas (OEPA) and the World Health Organization (WHO) use the prevalence of the infective stage of *O. volvulus* larvae in the black fly vectors as a major metric for determining whether or not transmission has been successfully interrupted in an endemic community [13,14]. The threshold used for declaring elimination is less than one *O. volvulus* per 2,000 female black flies per endemic community. At least 6,000 flies must be tested by Polymerase Chain Reaction (PCR-pool screening) in each endemic community to satisfy this standard [13-15]. To date, the collection of such large numbers of black flies has been problematic, as the primary method for collecting host-seeking black flies is the use of human landing collections [16-18]. This method involves stationing adult humans in areas of high *Simulium* densities and collecting black flies that attempt to land and blood-feed. Apart from the fact that this method has been a subject of criticism due to the potential risk of human collector’s exposure to infection during the early stages of a control program [19], it may be difficult for fly collectors to capture the large number flies needed to document that transmission has been interrupted where biting rates are low or highly seasonal. Thus, the development of trap(s) to replace human landing collections is becoming increasingly important as the focus in onchocerciasis shifts from control to elimination, and in some cases post-treatment surveillance [20].

Various attempts have been made to design black fly traps using both visual and chemical attraction techniques [21-25]. Most of these studies were conducted in West Africa where members of the *Simulium damnosum* species complex serve as the primary vectors for the causative agent of onchocerciasis, *Onchocerca volvulus*. In Latin America, *Simulium ochraceum* sensu lato is the most important vector of this parasite; until recently it was associated with ≥ 70% of transmission in the six countries where the disease occurred. The ecology and behavior of *S. ochraceum* s.l. is distinctly different from *S. damnosum* s.l., the primary vector in Africa [26-29], potentially complicating the prospects for development of a single trap that efficiently captures both species. As part of longitudinal studies in designing an improved black fly trap for monitoring and surveillance transmission of *O. volvulus*, field studies were carried out at endemic sites in Chiapas, Mexico, to evaluate different trap designs. Here we report the results of field trials and optimization of a promising design (Esperanza window trap) for the collection of host-seeking *S. ochraceum* s.l.

## Materials and Methods

### Ethics Statement

All procedures involving human landing collections were reviewed and approved by the appropriate institutional review boards of the countries involved. These included the Bioethics Committees of the Center for Research and Development in Health Sciences of the Autonomous University of Nuevo León (Monterrey, Nuevo León, Mexico), and an Institutional Review Boards of the University of South Florida. Written informed consent was obtained from all human landing collectors.

### Strategic design

**Figure 1:**
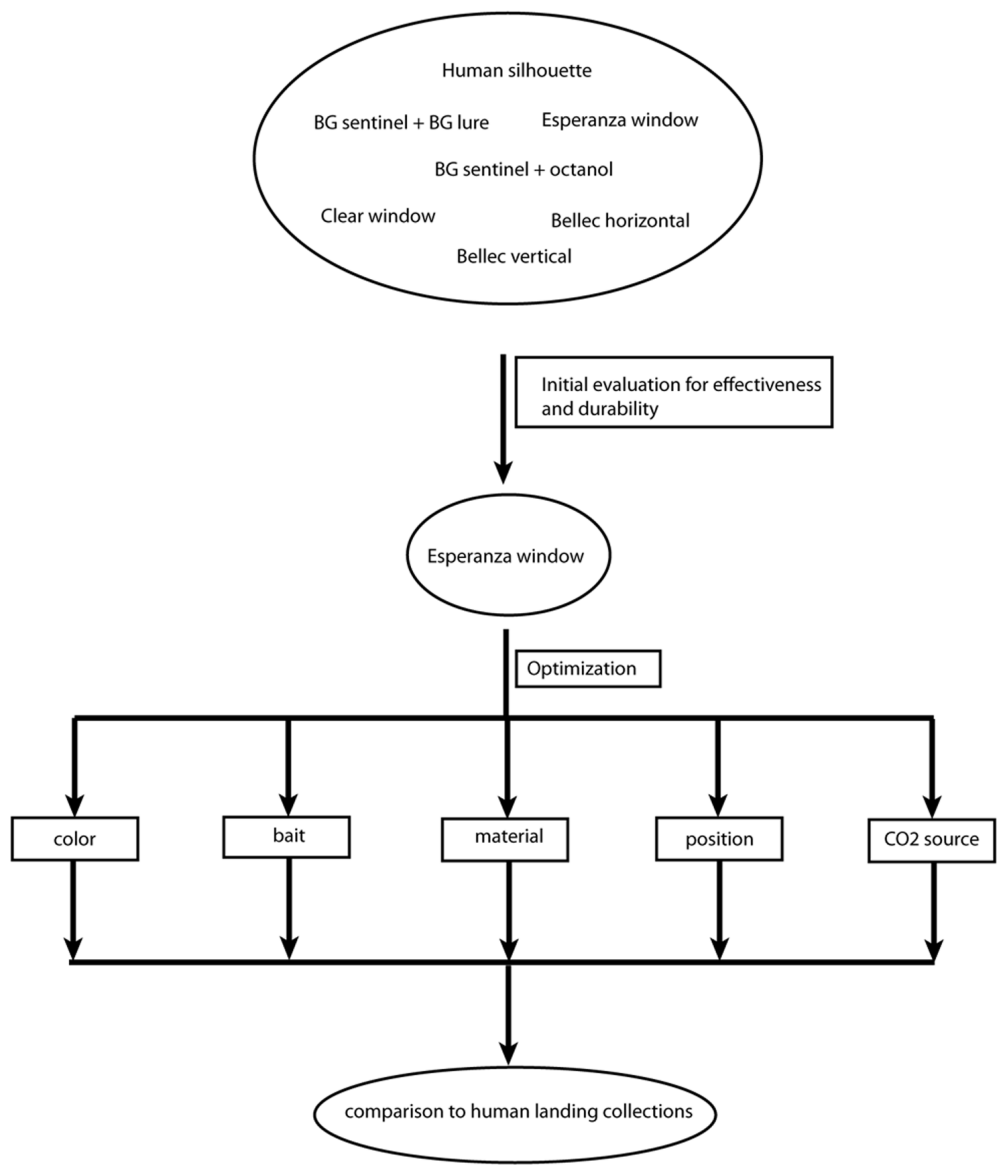
Strategic design of trap evaluation and optimization studies.

The strategy to design a trap to replace human landing collections is summarized in Figure 1. In the initial evaluations, diverse trap designs were compared, selecting those that showed initial promise and eliminating those that either collected few flies or were operationally impractical under field conditions. The platform showing the most promise in the initial evaluation was then optimized with different combinations of the factors that are known to contribute to trap effectiveness, especially with respect to visual and olfactory cues (trap color, olfactory lures). Collections from the optimized trap were compared to vector collectors employing a standard human landing collection protocol to estimate trap efficiency.

### Study area

All field studies were conducted in the village of Las Golondrinas, Chiapas, (15°25´ 59˝ N; 92°39´ 06˝ W, elevation 890 m) Mexico. Las Golondrinas is located within the former Southern Chiapas focus of onchocerciasis, where transmission of *O. volvulus* was quite high, prior to recent elimination of the infection [30]. Field studies were conducted during the dry season to take advantage of the consistently high populations of *Simulium ochraceum*, the primary vector of *O. volvulus* in the region [32]. Populations of *Simulium ochraceum* reach maximum levels during this time, with daily human landing rates often exceeding 100 over a 4-5 month period in certain locations. Las Golondrinas is a rural community on the forested Pacific Ocean-facing slopes of the Sierra Madre Mountains in habitat well suited for cultivation of coffee and cacao. Community leaders were consulted and approved the use of the selected locations in the village and on nearby riverbanks as vector catching points. Endangered species were not involved in this work.

### Trap evaluations against *S*. *ochraceum* s.l. in Chiapas, Mexico

#### Trap selection and first evaluation

**Figure 2:**
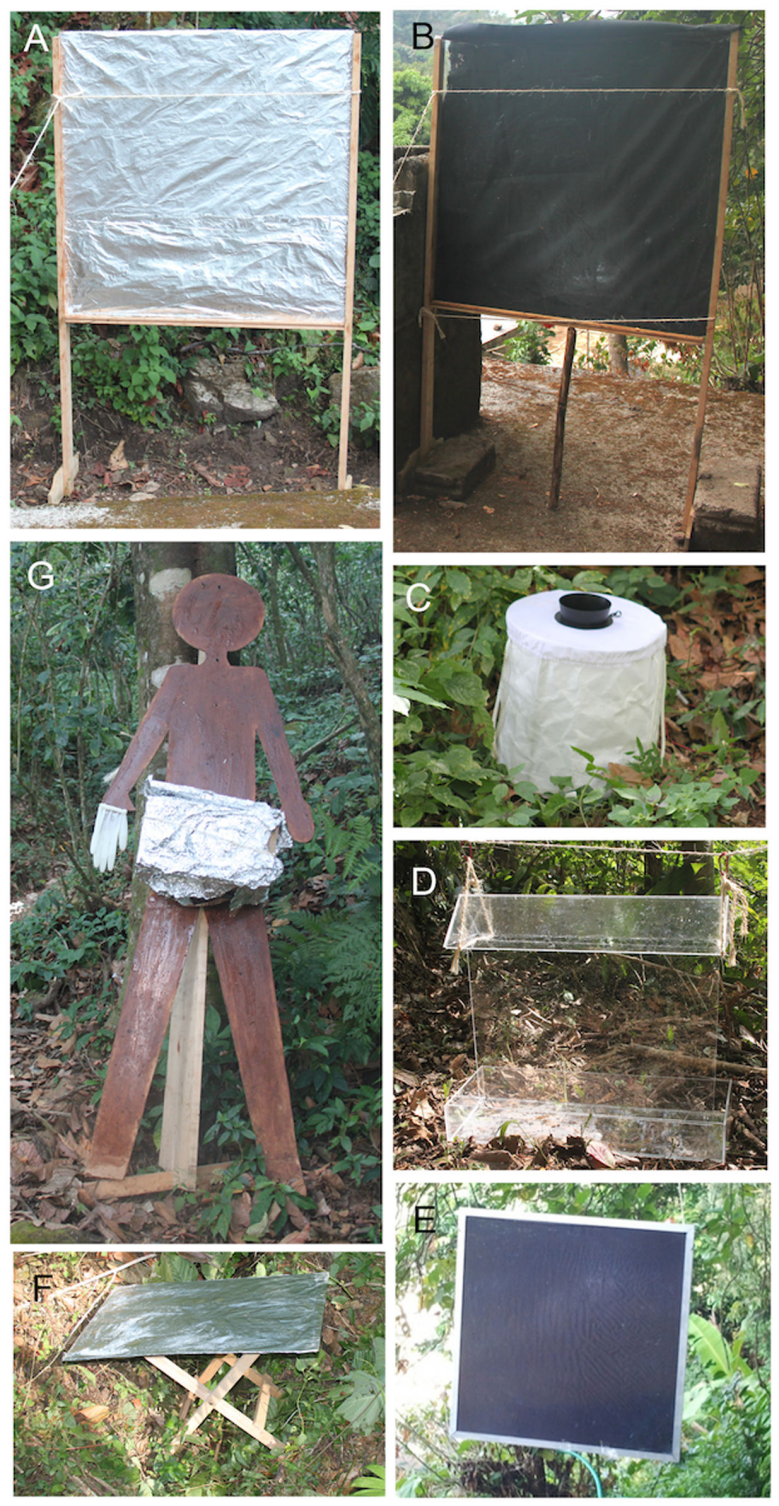
Traps evaluated for potential in collecting *Simulium ochraceum* s.l. females in Mexico. Panel A: vertical Bellec plaque. Panel B: Esperanza window trap (original). Panel C: BG Sentinel. Panel D: clear window trap. Panel E: Esperanza window trap (acrylic/hanging version). Panel F: horizontal Bellec plaque. Panel G: human silhouette trap (latex examination glove for scale).

Seven trap designs were initially selected for field studies, based on published reports and/or preliminary observations by the authors (Figure 2). These were (1), A human Silhouette: A 2-m high wooden human silhouette trap coated with Tangle-Trap Insect Trap Coating^®^ (The Tanglefoot Company, Grand Rapids, MI) and baited with BG-Lure**™** attractant (a mixture of compounds found in human skin secretions [Biogents AG, Regensburg, Germany]) (2), BG Sentinel with BG-Lure**™**: BG-Sentinel trap (Biogents AG, Regensburg, Germany) baited with BG-Lure**™** attractant (3) BG Sentinel with octenol: BG-Sentinel trap baited with 1-octen-3-ol lure (AgriSense Ltd, UK); (4) Clear window trap: a flight intercept trap [31] consisting of a 0.5 x 0.25 m sheet of clear acrylic (3 mm thickness) suspended vertically above a collection tray partially filled with a nontoxic polysorbate surfactant (~1.0% Tween-20® non-ionic detergent in water); (5) Bellec vertical: An oviposition trap [21] consisting of a 1.0 m^2^ piece of fiberboard (3 mm thickness) covered in aluminum foil and coated with a film of Tangle-Trap adhesive, and suspended 0.6 m above ground, oriented vertically; (6) Bellec horizontal: A 1.0 x 0.5 m piece of plywood (3 mm thickness) covered in aluminum foil and coated with Tangle-Trap adhesive, and supported 0.5 m above ground, oriented horizontally; and (7) “Esperanza window trap”: a novel design consisting of a 1m^2^ piece of black satin fabric sandwiched between two sheets of 1m^2^ glass (3mm thickness) that were each coated on their outer faces with Tangle-Trap adhesive. The appearance of the Esperanza window trap differed between sides (shiny and dull) depending on the orientation of the satin fabric. The Esperanza window trap was baited with BG-lure and supported 0.6 m above ground with a wooden frame. The traps were evaluated concurrently at two forested plots within the village, each proximal to an *S. ochraceum* breeding site (Figure S1). With the exception of the BG-Sentinel (a trap designed for collecting mosquitoes) and the Esperanza window trap, the traps deployed have been previously used for collecting *S. damnosum* s.l. in Africa [21-23,25]. The Esperanza window trap was initially developed and evaluated by the authors during preliminary studies at La Esperanza (Latitude 17°37′40″ N and Longitude 96°22′10″ W, elevation 1,600 m), a community in the state of Oaxaca, an area with a history of endemic onchocerciasis.

The traps were evaluated simultaneously at two forested plots within the village for six consecutive days, with trap position rotated daily to avoid position bias. A small intermittent stream flowed through each plot, serving as a breeding site for *S. ochraceum* (data not shown). Seven trap positions were established at each plot, in a rough circle with the stream at its center. Trap positions were 10-20 m apart and 2-5 m from the stream. Collections were retrieved three times daily (1100 h, 1400 h, and 1700 h). Flies collected between 11:00 h and 14:00 h in each trap were dissected for parity and gravidity.

#### Optimization of Esperanza window trap

**Figure 3:**
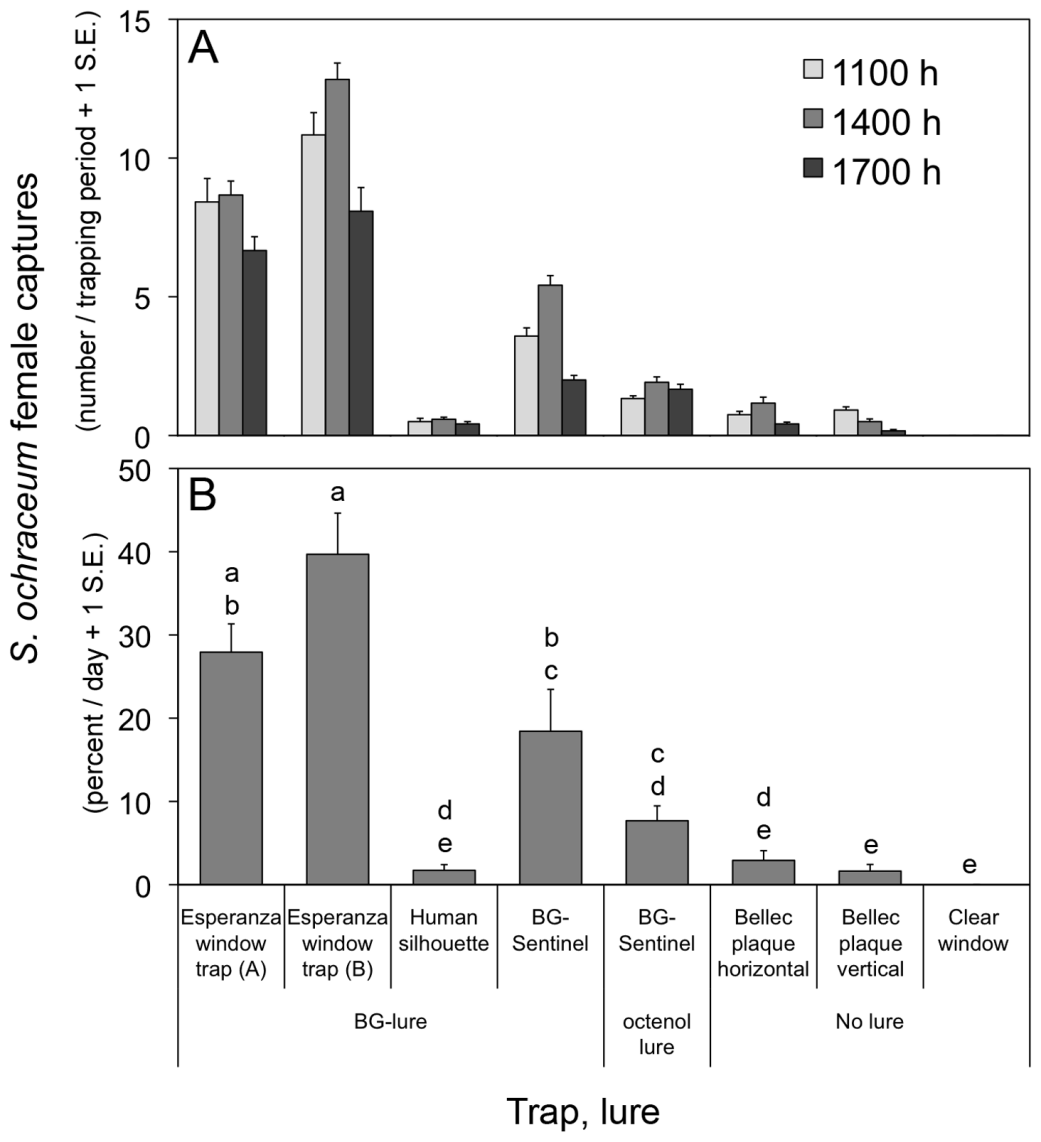
Comparison of traps for collecting the onchocerciasis vector *Simulium ochraceum* s.l. in the community of Las Golondrinas, Chiapas in Mexico. Traps were operated simultaneously, with positions rotated daily. Panel A: Mean females captured at each sampling time (1100 h, 1400 h, 1700h) over six days. Panel B: Percentage of total females captured each day by traps over six days. Letters denote significant differences among traps for the percentage of total flies captured each day.

Following the initial trials that resulted in the selection of the Esperanza window trap as the most promising design, the Esperanza window trap was modified from the original prototype to increase its ease of use and its overall effectiveness (Figure 2, Panel E). Modifications included (1) replacing glass panes with acrylic sheeting (2 mm thickness), strengthening the trap and permitting easy movement of the trap in the field; (2) replacing the wooden stand with an aluminum frame, suspended from a tree branch (rather than standing on ground) (Figure 2); (3) evaluation of CO_2_ (two sources), octenol and worn shirts containing human secretions as potential attractants; (4) different colors of fabric; and (5) comparison with vector collectors (human bait).

The original model of the Esperanza window trap was first tested against one with acrylic panes (instead of glass) and against a human landing collection. The traps were arranged in an array roughly 7 m from the breeding site, separated by a distance of 3 m from one another. Human landing collections were carried out approximately 5 m from the traps. These distances between traps and between traps and human landing collections minimized the interference among traps while still drawing from the same host-seeking black fly population. This arrangement permitted flies to choose amongst traps (including human bait), putting all the traps in competition. Human landing collections were carried out with two persons working as a team: one person with exposed upper body, while the other person collected the flies landing on the exposed skin of his colleague, as previously described [7]. The fly collector’s duties were rotated every counting period (180 minutes) to minimize the effect of individual attraction to the flies. Before the commencement of every trapping session, the traps were checked closely to remove any black flies that were trapped during non-experimental periods.

The acrylic-paned Esperanza window traps were then used in field trials to determine which chemical attractants could be employed to increase collections of *S. ochraceum* s.l. to levels rivaling that of vector collectors. Treatments included CO_2_ alone; CO_2_ plus octenol; CO_2_ plus BG-lure; CO_2_ plus a shirt worn by a farm worker for three consecutive days (without washing); ethanol (5mL dispensed from perforated plastic screw-cap vial); and completely unbaited. Traps were placed in a line roughly 7m from an *S. ochraceum* breeding site, and 3m from one another. The CO_2_ baited traps were connected to the source of CO_2_ (gas cylinders) with flexible tubing (Figure 2). The gas was released at each trap at a rate of 150-200 mL/min. Due to the limited supply and high dispense rate of CO_2_, it was not feasible to operate the traps throughout the entire day. Therefore, the traps were operated for 20 minutes per hour between 800 h and 1200 h, targeting the period of highest biting of *S. ochraceum* s.l. [32]. Trap positions were rotated daily, and trials were conducted four times daily for three consecutive days. Human landing collections were carried out as described above, at the same time and for the same duration (20-minute periods) as the trap evaluations.

The effectiveness of yeast-generated CO_2_ [33,34], as an alternative to commercial CO_2_, was investigated simultaneously with different colors of fabric [35]. Esperanza window traps, with black, blue, red or yellow fabric, and yeast-generated CO_2_, were evaluated over three days at the same plots as above. Fabric for the traps was obtained from Grupo Parisina S.A. de C.V. (Mexico City, MX). The product numbers for each fabric are as follows: Yellow 4917L72; Blue 4917L27; Black 4917L19; Red 4917L36. Descriptive colorimetric values for each fabric are provided in Table 1. Traps were operated for 20 minutes per hour between 800 h and 1200 h to conserve the limited supply of CO_2_.

**Table 1:** Colorimetric values of four nominal colors (red, yellow, blue, black) evaluated in Esperanza window traps in Chiapas, Mexico.

System	Red	Yellow	Blue	Black
RGB	rgb(225, 48, 54)	rgb(249, 191, 84)	rgb(52, 85, 178)	rgb(23, 21, 22)
Hex	#e13036	#f9bf54	#3455b2	#171516
CIELab	L: 49.963, a: 66.294, b: 40.651	L: 80.762, a: 10.162, b: 60.278	L: 38.623, a: 20.177, b: -53.084	L: 7.013, a: 1.162, b: -0.331
Hunter-Lab	L: 42.881, a: 61.387, b: 22.672	L: 76.185, a: 5.61, b: 40.516	L: 32.313, a: 13.611, b: -57.134	L: 8.811, a: 0.104, b: 0.324
CMYK	cyan: 0, magenta: 0.787, yellow: 0.76, key: 0.118	cyan: 0, magenta: 0.233, yellow: 0.663, key: 0.024	cyan: 0.708, magenta: 0.522, yellow: 0, key: 0.302	cyan: 0, magenta: 0.087, yellow: 0.043, key: 0.91

### Statistical analysis

The statistical differences among effectiveness of different traps and their lures were evaluated using a t-test (for 2 treatments) or ANOVA (for 3 or more treatments). For the initial trap evaluations, ANOVA was first used to test for differences in the number of flies collected at different sampling periods for each trap type. If no significant differences were found, data from the three sampling periods were aggregated. Since trap-to-trap interference may violate the independence of samples assumption of ANOVA, we used the Kruskal-Wallis test to test for significant differences among traps for black fly captures and the Tukey test for *post hoc* multiple means comparison. All tests were performed using SAS 9.1.3 (SAS Institute, Cary, NC, USA). Since day-to-day variation in fly activity was substantial and relative performance of traps was more relevant than absolute performance, for the purposes of statistical analysis raw counts were converted to proportions (of total flies collected during a given period and plot). In the first evaluation of the Esperanza window trap in Mexico, counts from each face of the trap were analyzed separately, since each side of the fabric had a different visual quality (shiny vs. dull). Prior to statistical tests, all proportions were transformed using the angular transformation (arc sine of square root).

## Results

### Initial trap evaluations

Significant differences in the number of *S. ochraceum* collected were observed among the different trap platforms that were initially evaluated at the field locations in Chiapas, Mexico (H=158.56; d.f.=7; P < 0.001, Kruskal–Wallis test). The Esperanza window trap and BG Sentinel traps consistently outperformed other traps, regardless of time of day (Figure 3). The number of females collected at 1100 h, 1400 h, and 1700 h was not significantly different for any trap type (Table 2). Collections on the two faces (shiny or dull) of the Esperanza window trap were also not significantly different (Figure 3). Other black fly species were also collected, namely *S*. *metallicum* s.l. and *S. gonzalezi*, but their numbers were low in comparison to those of *S. ochraceum* s.l. (data not shown).

**Table 2:** Variation in *Simulium ochraceum* females captured by traps at three sampling times (1100 h, 1400 h, 1700h) in Chiapas, Mexico.

	Flies per collection period				
Trap	1100 h	1400 h	1700 h	*F*	*P*
Esperanza window trap (both sides)	19.25	21.50	14.75	0.59	0.5577
Human silhouette (BG-lure)	0.50	0.58	0.42	0.06	0.9379
BG- Sentinel (BG-lure)	3.58	5.42	2.00	3.1	0.0585
BG- Sentinel (octenol lure)	1.33	1.92	1.67	0.27	0.7661
Bellec plaque horizontal	0.75	1.17	0.42	0.54	0.5857
Bellec plaque vertical	0.92	0.50	0.17	1.41	0.2578
Clear window trap	0.00	0.00	0.00	nd	nd

### Optimization of the Esperanza window trap

**Figure 4:**
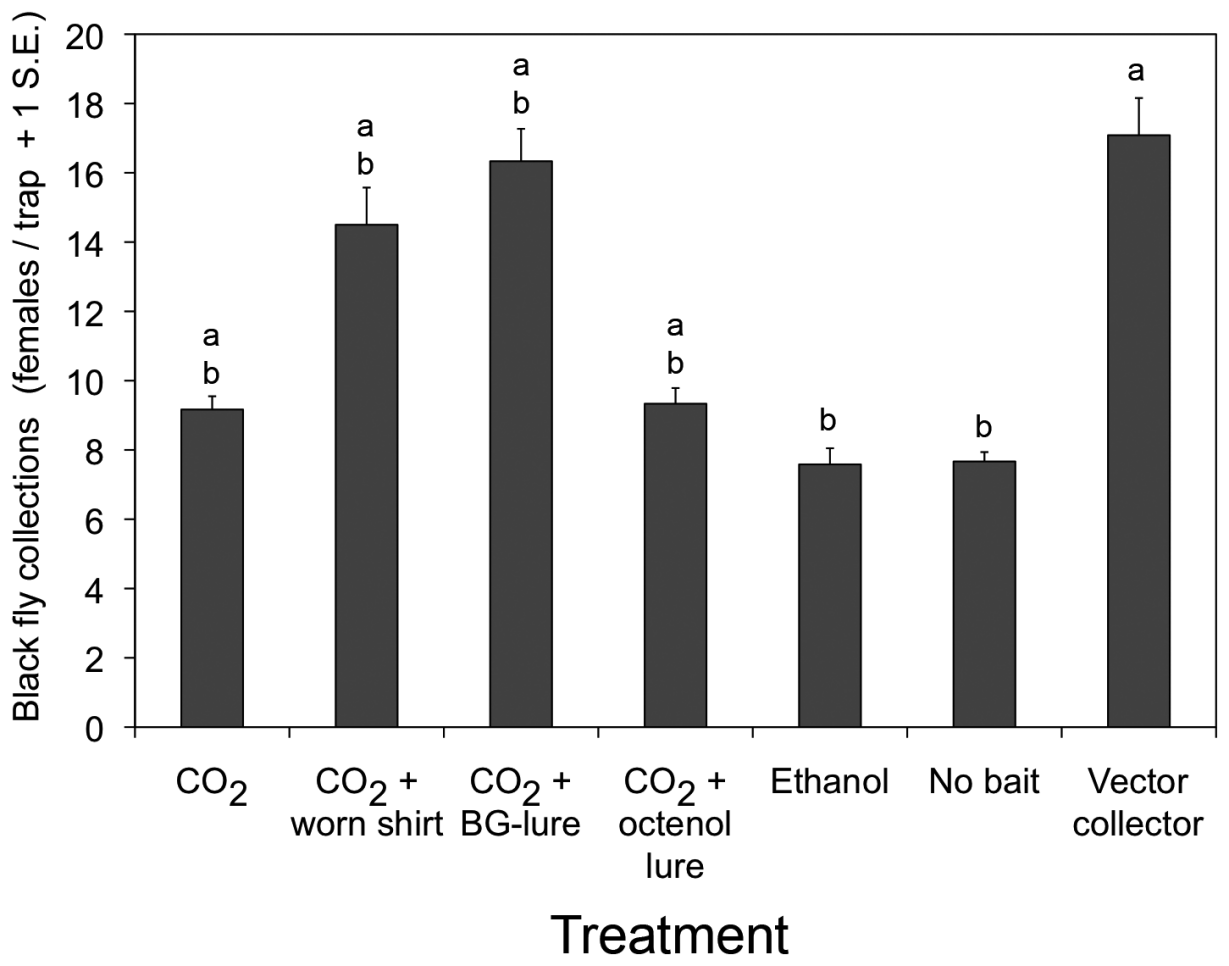
Comparison of baited and unbaited Esperanza window traps and vector collectors for collecting *Simulium ochraceum* s.l. females. Treatments included CO_2_ (commercial grade), a shirt worn by a local villager, BG-lure, 1-octen-3-ol, ethanol (control), unbaited trap, and a vector collector team. Data are from 20-min collecting periods, at a site in the community of Las Golondrinas, Chiapas, Mexico, 2012. Letters denote significant differences among treatments for the percentage of total flies captured each day.

**Figure 5:**
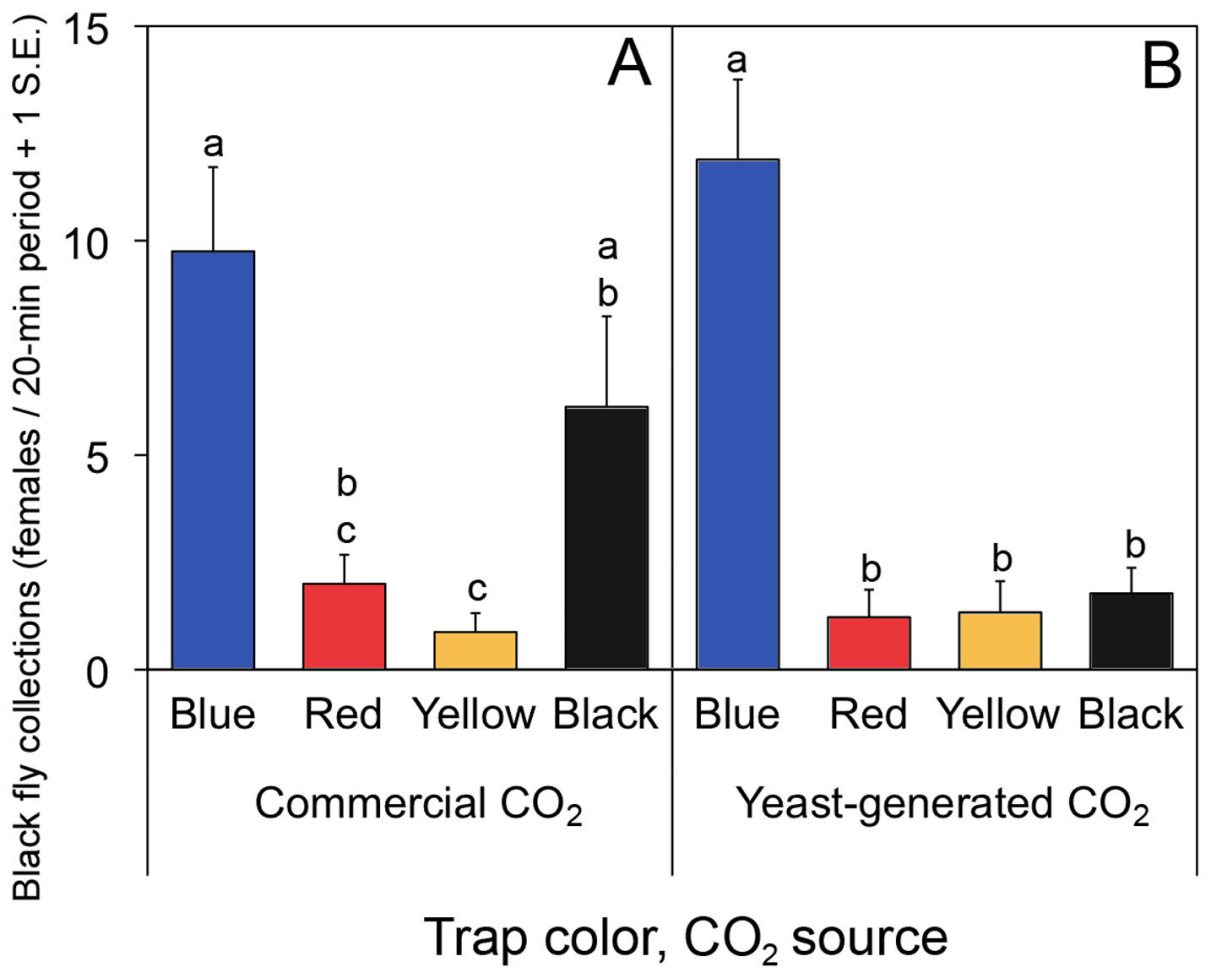
Evaluation of trap color and CO_2_ source of Esperanza window traps for collecting *Simulium ochraceum* s.l. females. All traps were co-baited with the BG-lure. Data are from 20-min collecting periods in the community of Las Golondrinas, Chiapas. Letters denote significant differences among traps for the percentage of total flies captured each day. Panel A: Esperanza window traps baited with commercial CO_2_. Panel B: Esperanza window traps baited with yeast-generated CO_2_.

**Figure 6:**
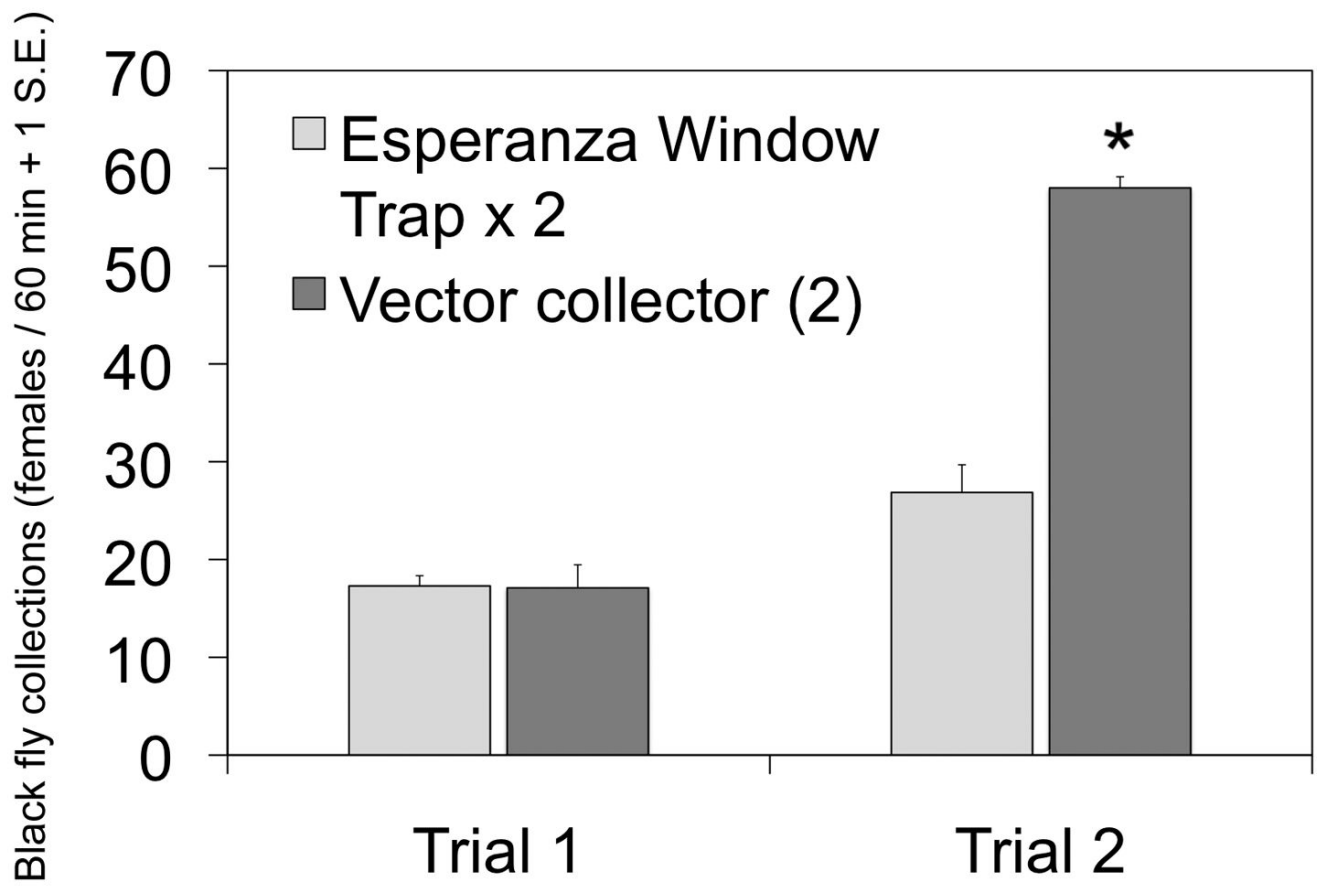
Comparison of Esperanza window traps and vector collectors for collecting *Simulium ochraceum* s.l. females. Traps were baited with CO_2_ (yeast-generated) and BG-lure. Data are from 60-min collecting periods, during two trials at plots in the community of Las Golondrinas, Chiapas, Mexico. Asterisks denote significant differences among treatments for the percentage of total flies captured each day, as determined by t-test.

Optimization of the Esperanza window trap revealed that significant improvements and modifications of the original (prototype) design were feasible, facilitating deployment and increasing catches. Esperanza window traps with acrylic panes mounted in aluminum frames were far easier to handle and collected significantly more black flies (T=9.06, p<0.001) than those fitted with glass panes in wooden frames (acrylic: daily mean=52.7, s.d.=11.7; glass: daily mean=32.3, s.d.=4.45). Initial trials of various chemical lures showed that Esperanza window traps baited with CO_2_ and human scent (a worn shirt or BG-lure) could approach the attractiveness of a human landing collection (Figure 4). Esperanza window traps without baits and those baited with control substances (ethanol alone) caught the fewest number of flies (Figure 4). Significant differences in the number of flies captured were found when different colors of fabric used in Esperanza window traps baited with yeast-generated (F_3,32_=19.30, p<0.001) or bottled carbon dioxide (F_3,32_=10.12, p<0.001), with blue fabric traps generally outperforming traps with other colors (Figure 5). Black fly collections of Esperanza window traps baited with yeast-generated CO_2_ were not significantly different from those baited with commercial CO_2_ (T=2.14; p=0.054) (Figure 5). When blue Esperanza window traps baited with BG-lure and yeast-generated CO_2_ were compared with vector collectors (2 individuals working together) in two trials, the results showed that the number of flies captured on two traps was not significantly different than that captured by a vector collector team in the first trial (T=-0.24; p=0.82), but was about half that captured vector collector team in the second trial (T=-6.82; p<0.001) (Figure 6). Dissection of the flies captured by the traps or collection team revealed that the parity rates in the flies collected by both methods were not significantly different, exceeding 95% in both populations (data not shown).

## Discussion

The data presented here demonstrate the feasibility of using traps to replace human bait (vector collectors) in onchocerciasis monitoring and elimination programs. In initial field trials it was obvious that the several trap designs evaluated were not equally effective for collecting *S. ochraceum* s.l. females, but that some traps would be amenable to further development and experimentation (e.g. Figure 3). The Esperanza window trap showed the most initial promise, and also proved to be effective in subsequent tests (Figures 4-6). Since repeatability of results has been lacking in most black fly trapping studies [36], the consistent productivity of the Esperanza window trap was very encouraging.

After the first round trap comparisons field tests focused solely upon optimizing the Esperanza window trap for collecting *S. ochraceum* s.l. females. Modifications to the Esperanza window traps included replacing glass with acrylic panes and replacing the awkward wooden frame with aluminum, resulting in a lighter, and more manageable design that collected more black flies than the initial prototype. Additional experiments testing the effectiveness of available chemical lures (including CO_2_) indicated that olfactory attractants were key in trapping success.

It is interesting that Esperanza window traps with blue fabric collected more *S. ochraceum* s.l. females than did traps with other colors of fabric. Blue is reported to be attractive to tsetse flies (*Glossina* spp.) [37], and it is used as a dominant fabric color in tsetse trap fabrication [38]. Yeast-generated CO_2_ was as effective as commercial CO_2_. This is particularly important since onchocerciasis-endemic areas are often quite remote, and obtaining CO_2_ cylinders in these areas can pose a serious operational difficulty. Yeast-generated CO_2_ will likely conserve monetary resources, since the ingredients (yeast, sugar, water) and materials (plastic bottles, plastic tubing/hose) are easy to locate or improvise. Furthermore fermentation of sugar by yeast produces not only CO_2_, but also a variety of volatile organic compounds (alcohols, esters, aldehydes and fatty acids) that are known to attract the arthropods that are attracted to vertebrates [33].

Simultaneous testing of multiple trap types, as performed in our initial trap screening presents several operational and analytical challenges. Some of the traps tested present large visual cues (Bellec plaques and Esperanza window traps), while others presented primarily olfactory cues (e.g. BG-Sentinel). The Esperanza window trap presented a large visual target as well as olfactory lures. Testing this broad array of trap types, some with olfactory cues and others without makes it quite difficult to tease apart the subtle interplay of long and short-distance stimuli that ultimately result in a fly being captured by a particular trap. For example, a long distance olfactory cue emanating from one of the trap types might draw flies into the field plot. Once in the vicinity of the traps, a shorter distance signal (visual or olfactory) from a different trap type may cause a change in a fly’s trajectory so that the fly is actually captured by a trap that is different from the one that originally lured the fly to the experimental plot. In this scenario, the trap that ends up capturing the fly may perform better in the presence of a long-distance olfactory cue originating from a neighboring trap than if evaluated without other traps. However, given the temporal (day-to-day), spatial and environmental variation in fly activity, testing traps independently (on different days) and then trying to compare trap captures between traps across different days or locations would present its own set of analytical challenges. That said, our results indicate that the traps that utilized chemical lures caught the overwhelming majority of black flies (Figure 3). The four traps that utilized lures (Esperanza window trap, human silhouette, BG-Sentinel with human scent lure and BG-Sentinel with octenol) collected 95.4% of the captured flies, while the three unbaited traps (Bellec plaques horizontal and vertical and Clear window trap) captured only 4.6% of the total. This would suggest that flies are drawn into the trapping arena by olfactory cues emitted by baited traps, but traps with multimodal profiles, such as the Esperanza trap, outperformed unbaited traps in actually capturing flies.

Exactly what features of the Esperanza trap are responsible for its relative “success” in the field are not easy to ascertain. The optimized version of the trap utilized multiple visual and olfactory cues that likely worked in concert to maximize its attractiveness to *S. ochraceum* s.l. females. Trap color appeared to have a strong effect on black fly capture, when other variables were held constant. Experimental evidence suggests that spectral reflectance and polarization of reflected light are key determinants of how insects respond to visual stimuli [39]. For example, the black-and-white striped patterns of zebra coats result in alternating directions of reflected light polarization. Stripe width has a profound impact on attractiveness of zebras to tabanids, with attraction decreasing along with decreasing stripe width, due to disruptive patterns of reflected light polarization [40]. Spectral reflectance of visual stimuli (especially host silhouettes) is thought to be inversely related to attraction of host-seeking diurnal flies, including black flies [41] and tabanids [42]. The number of simuliids orienting and landing on silhouette traps of various reflectances was found to be greater when reflectance was lowest [41]. Tabanid traps treated with a product that reduced ultraviolet reflectance from cloth fabrics increased the catch of tabanids in canopy traps by 24% [42]. The optimized Esperanza window trap with clear acrylic panels (nearly eliminating UV reflectance [43]) collected significantly more females than the trap with glass panels, supporting the notion that spectral reflectance is inversely related to attractiveness of a visual stimulus to black flies. Though only a few hues (of the virtually limitless variety of colors) were evaluated in the current study, the results obtained here generally corroborate the findings of others – that blues and blacks are attractive to host-seeking simuliids [41].

Esperanza window traps, baited with olfactory attractants and CO_2_, were nearly as productive as a vector collector team (2 adults working together) in some trials. Considerable variation in trap effectiveness might be expected, based on site-specific conditions. Esperanza traps should therefore be evaluated at other locations with *O. volvulus* vectors to determine their effectiveness relative to human landing collections. However, it is probably not necessary that an effective trap be as productive as a vector collection team, since a single person can easily maintain five traps (data not shown). By deploying multiple traps, a person working alone can increase daily fly captures by at least threefold over those obtainable using standard collection methods.

While the data presented above suggest that the Esperanza window trap shows promise as a substitute for human landing collections, additional work will be necessary before they can be employed for monitoring and surveillance of onchocerciasis elimination. First, the bait used in the traps must be standardized and optimized. The BG-lure, which is a commercial product, successfully attracted *S. ochraceum* in fairly large numbers. However, the BG-lure, which employs ammonia, lactic acid, and caproic acid as active ingredients [44], was designed and optimized to attract *Aedes* spp. mosquitoes [45]. It is likely that this mixture of compounds was not an optimal attractant for black flies and that inclusion or substitution of other compounds found in human breath and sweat will result in an improved bait formulation.

Second, the models used to identify transmission thresholds for *O. volvulus* have all relied upon entomological indicators derived from human landing collections. Thus, it will be necessary develop algorithms to relate the trap collections to human biting rates and infective rates. In this regard, the fact that the traps appear to collect fly populations that exhibit parity rates which are not significantly different from those obtained by human landing collections carried out in parallel will simplify this process. Previous studies that have examined variability of infection rates associated with human landing collections in *S. ochraceum* s.l. [46] may prove to be a useful foundation in developing methods to relate trap collections to infective biting rates.

In conclusion, the Esperanza window trap, when baited with CO_2_ and olfactory lures collected substantial numbers of *S. ochraceum* s.l., one of the principal vectors of *O. volvulus* in Latin America. This trap has the potential for replacing humans as bait in the monitoring and surveillance of onchocerciasis vectors. Eventually, such a trap may prove to be an effective replacement for vector collectors in areas of Latin America and Africa where onchocerciasis remains endemic. These traps have the potential to be extremely useful tools in aiding in the certification of elimination of *O. volvulus* transmission and in the post-treatment surveillance era. 

## Supporting Information

Figure S1 **Overhead view of a field plot: The photo includes a portion of one of the plots used to evaluate the trap types.** A Bellec plaque and Esperanza window trap are visible.
(TIF)
