# Supplementary figures and images for: Development of a Novel Trap for the Collection of Black Flies of the Simulium ochraceum Complex
Source: PLoS One. 2013 Oct 7;8(10):e76814. doi: 10.1371/journal.pone.0076814 (PMC3792067; doi:10.1371/journal.pone.0076814)

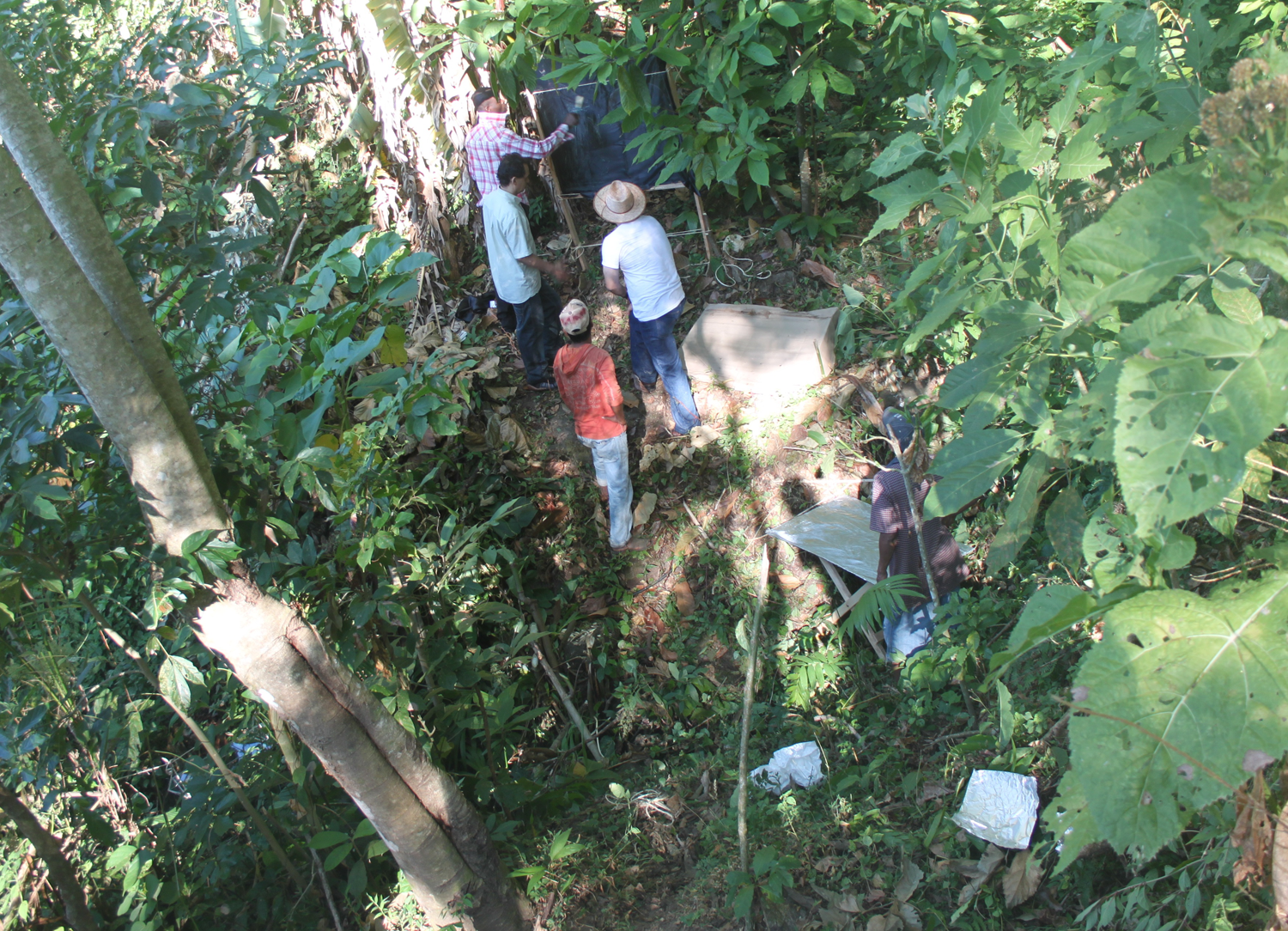

Supplement: Figure S1 — Overhead view of a field plot: The photo includes a portion of one of the plots used to evaluate the trap types. A Bellec plaque and Esperanza window trap are visible. (TIF) [file pone.0076814.s001.tif]
